# Sleep Duration, Sleep Quality, Body Mass Index, and Waist Circumference among Young Adults from 24 Low- and Middle-Income and Two High-Income Countries

**DOI:** 10.3390/ijerph14060566

**Published:** 2017-05-26

**Authors:** Karl Peltzer, Supa Pengpid

**Affiliations:** 1 HIV/AIDS/STIs and TB (HAST), Human Sciences Research Council, Pretoria 0001, South Africa; 2 Department of Research & Innovation, University of Limpopo, Sovenga 0727, South Africa; supaprom@yahoo.com; 3 ASEAN Institute for Health Development, Mahidol University, Salaya, Phutthamonthon, Nakhon Pathom 73170, Thailand

**Keywords:** sleep duration, sleep quality, body mass index, waist circumference, young adults, low- and middle-income countries

## Abstract

Obesity and its comorbidities have emerged as a leading public health concern. The aim of this study was to explore the relationship between body mass index (BMI), waist circumference (WC) and sleep patterns, including duration and disturbances. A cross-sectional questionnaire survey and anthropometric measurements were conducted with undergraduate university students that were randomly recruited in 26 universities in 24 low- and middle-income and two high-income countries. The sample included 18,211 (42.1% male and 57.9% female, mean age 21.0 in male and 20.7 years in female students) undergraduate university students. The overall BMI was a mean of 22.5 kg/m^2^ for men and 22.0 kg/m^2^ for women, and the mean WC was 78.4 cm for men and 73.8 cm for women. More than 39% of the students reported short sleep duration (≤6 h/day) and over 30% reported moderate to extreme sleep problems. In a linear multivariable regression, adjusted for sociodemographic and lifestyle factors, short sleep duration was positively associated with BMI in both men and women, and was positively associated with WC among women but not among men. Sleep quality or problems among men were not associated with BMI, while among women mild sleep problems were inversely associated with BMI, and poor sleep quality or problems were positively associated with WC both among men and women. The study confirmed an association between short sleep duration and increased BMI and, among women, increased WC, and an association between poor sleep quality and increased WC but not BMI. Further, differences in the association between sleep characteristics and BMI and WC were found by region and country income.

## 1. Introduction

Globally, the adult (≥18 years) prevalence of overweight and obesity (Body Mass Index = BMI ≥ 25 kg/m^2^) was 39% and 13%, respectively, in 2014 [1]. In predominantly low- and middle-income regions, the following prevalence of overweight and obesity was found: In the Central Latin American and Caribbean region, the adult (≥20 years) prevalence of overweight and obesity was 47.5% among men and 57.8% among women; in the sub-Saharan Africa region 2 it was 6.6% among men and 36.9% among women; in the South Asia and Southeast Asian region it was 21.2% among men and 25.4% among women; and in Central Asia, North Africa and Middle East and Eastern Europe it was 55.4% among men 58.8% among women (the numbers reported are derived from the original numbers) [2]. Compared with the global prevalence of overweight and obesity, the Central Asia, North Africa and Eastern European region and the Central American and Caribbean region had a higher prevalence of overweight and obesity than the global prevalence, while the sub-Saharan Africa and South Asia and Southeast Asian regions had a lower prevalence overweight and obesity than the global prevalence.

Obesity is a major risk factor for a number of non-communicable diseases, such as “diabetes mellitus, cardiovascular disease, hypertension and stroke, and certain forms of cancer” leading to the “increased risk of premature death to serious chronic conditions that reduce the overall quality of life” [3]. Interventions to prevent and mitigate the global obesity burden are needed [4,5]. There is evidence that “diet-induced body-weight loss and successive body-weight maintenance contribute to sleep improvement” [6]. A recent study [7] investigated whether “enhancing children’s sleep may show promise in assisting with weight regulation.” Various reviews of cross-sectional and longitudinal studies have found evidence that short sleep duration and poor sleep quality are associated with general obesity and central obesity [8,9,10,11].

Cross-cultural studies within low- and middle-income countries and across different world regions are lacking, however, they important in identifying the links between sleep and obesity patterns [12]. This may be especially important among young adults, including university students, because of accompanying lifestyle changes such sleep patterns, as well as dietary and physical activity behaviour taking place in the transition from home to university [13]. This may help in distinguishing obesity factors determined by biological and socio-environmental factors, which are needed in culturally appropriate obesity prevention strategies [12]. It is hypothesised that short sleep duration and poor sleep quality will be associated with increased BMI and increased waist circumference (WC).

The aim of the study was to investigate cross-cultural differences in the association between sleep duration, sleep quality, BMI, and WC among young adults from 24 low- and middle-income and two high-income countries.

## 2. Materials and Methods

### 2.1. Sample and Procedure

This was a cross-sectional study using a self-administered questionnaire and anthropometric assessment in randomly selected undergraduate university students from conveniently selected 26 universities in 26 countries: Central Latin America: Colombia (*n =* 811), Venezuela (*n =* 444), and Caribbean: Barbados (*n =* 576), Grenada (*n =* 235), Jamaica (*n =* 676); sub-Saharan Africa: Ivory Coast (*n =* 781), Kenya (*n =* 820), Madagascar (*n =* 779), Mauritius (*n =* 450), Namibia (*n =* 468), Nigeria (*n =* 804), South Africa (*n =* 764); *South Asia*: Bangladesh (*n =* 648), India (*n =* 794), Pakistan (*n =* 753), and Southeast Asia: Laos (*n =* 758), Malaysia (*n =* 1023), Myanmar (*n =* 328), Philippines (*n =* 766), Singapore (*n =* 677), Thailand (*n =* 782), Vietnam (*n =* 764); Central Asia: Kyrgyzstan (*n =* 813); North Africa and Middle *East*: Tunisia (*n =* 917), Turkey (*n =* 794), and Eastern Europe: Russia (*n =* 786). We have grouped the above regions into four regions, as follows: (1) Central Latin America and the Caribbean, (2) Sub-Saharan Africa, (3) South and Southeast Asia = South Asia and Southeast Asia, and (4) Central Asia and neighbouring regions = Central Asia, North Africa and Middle East, and Eastern Europe.

The anonymous, self-administered questionnaire used for data collection was developed in English, then translated by two independent bilingual translators into Arabic, French, Lao, Myanmar, Russian, Spanish, Thai, Turkish and Vietnamese. Another bilingual translator, who had no knowledge of the original instrument, back-translated the reconciliated target language version. In cases where a translated version of specific sections of the questionnaire, for example, the International Physical Activity Questionnaire, was available, this was not translated a second time.

Research assistants working in the participating universities asked classes of undergraduate students to complete the questionnaire at the end of a teaching class, followed by the anthropometric measurements. In each study country, undergraduate students were surveyed in classrooms selected through a cluster sampling procedure. A university department formed a cluster and was used as a primary sampling unit. One department was randomly selected from each faculty. For each selected department, undergraduate courses offered by the department were randomly ordered. The students who completed the survey varied in the number of years for which they had attended the university. A variety of majors were involved, including education, humanities and arts, social sciences, business and law, science, engineering, manufacturing and construction, agriculture, health and welfare and services. The universities involved were located in the capital cities or other major cities in the participating countries, as detailed in the acknowledgement.

Informed consent was obtained from participating students, and the study was conducted from 2013 to 2015. Individual participation rates were in all countries more than 90%, except for Indonesia (69%) and Myanmar (73%). Ethics approvals for the study protocol were obtained from all participating institutions.

### 2.2. Measures

The outcome measures are BMI and waist circumference, the independent variables are sleep duration and sleep quality, and the confounding variables include sociodemographic factors (age, gender, and socioeconomic status) and life style factors (tobacco use, binge alcohol use, physical inactivity, sedentary behaviour, and depressive symptoms).

Anthropometric measurements: Students’ anthropometric measurements were recorded by trained researchers using standardised protocols [14]. Standing height was measured to the nearest 0.1 cm without shoes, using a stadiometer. Participants, wearing light clothes, were weighed to the nearest 0.01 kg on a load-cell-operated digital scale which was first calibrated using a standard weight and re-checked daily [15]. Waist circumference (WC) was measured around the waist through a point one third of the distance between the xiphoid process and the umbilicus, using a non-stretchable tape measure [16]. Body mass index (BMI) was calculated as weight in kg divided by height in metres squared.

Sleep duration: Students were asked, “On average, how many hours of sleep do you get in a 24 h period?” [17]. Responses were divided into three categories: short sleep (≤6 h), reference category (7–8 h), and long sleep (≥9 h) [17,18].

Sleep quality was estimated based on the question: “Overall in the last 30 days, how much of a problem did you have with sleeping, such as falling asleep, waking up frequently during the night, or waking up too early in the morning?” Response options were: 1 (none), 2 (mild), 3 (moderate), 4 (severe), or 5 (extreme/cannot do). The last two categories were collapsed due to few responses of “extreme/cannot do” [19].

Sociodemographic questions included age, gender, and year of study. Socioeconomic background was assessed by relative family income within each country, identified as wealthy (within the highest 25%), quite well off (within the 50 to 75% range), not very well off (within the 25 to 50% range), or quite poor (within the lowest 25%); students were classified as wealthy if they considered themselves as wealthy or quite well off and poor if they considered themselves as coming from a not very well off or quite poor family background [20].

Tobacco use was assessed with the question: “Do you currently use one or more of the following tobacco products (cigarettes, snuff, chewing tobacco, cigars, etc.)?” (Yes, No). [21].

Binge alcohol use was measured by asking participants: “How often do you have (for men) five or more and (for women) four or more drinks on one occasion?” Response options were 0 = never, 1 = less than monthly, 2 = monthly, 3 = weekly and 4 = daily or almost daily; responses were dichotomised into 0 = never and 1 = less than monthly to daily or almost daily [22].

Physical activity was assessed using the self-administered International Physical Activity Questionnaire (IPAQ) short version, for the last seven days (IPAQ-S7S). We used the instructions given in the IPAQ manual for reliability and validity, which are detailed elsewhere [23]. We categorised physical activity (short form) according to the official IPAQ scoring protocol [24] as low, moderate, or high. Physical inactivity was classified as low physical activity.

Sitting time was measured with the IPAQ short form [24]: “During the last 7 days, how much time did you usually spend sitting on a weekday? Include time spent at work, at home, while doing course work and during leisure time. This may include time spent sitting at a desk, visiting friends, reading, or sitting or lying down to watch television” [24]. The question was answered in terms of hours and minutes. The average sitting time per day in minutes was calculated, and 480 min (8 h) was classified as high sitting time or sedentary behaviour [25].

Centres for Epidemiologic Studies Depression Scale (CES-D): We assessed depressive symptoms using the 10-item version of the CES-D and used a cut off score of 15 or more for severe depressive symptoms [26] (Cronbach alpha = 0.72).

### 2.3. Data Analysis

The data were analysed using STATA 13.00 (StatCorp LP, College Station, TX, USA). Descriptive statistics were used to describe the dependent variables, mean and standard deviations (SD) for BMI and WC, and independent variables, in percent for sleep variables as well as sociodemographic and lifestyle factors, including depression. A series of linear regressions were performed, with adjusting for sociodemographic and lifestyle factors, to test the relative contribution of sleep duration (short, normal, long) and sleep quality (mild, moderate, severe, or extreme sleep problems) on BMI and WC (as continuous variables), for men and women, low- or lower middle-income countries and upper middle- or high-income countries, and four regions (Central Latin America and Caribbean, sub-Saharan Africa, South/Southeast Asia and Central Asia and others), separately. Country income was classified according to the World Bank criteria into low-income countries (Gross National Income = GNI per capita of $1025 or less), lower middle-income countries (GNI per capita between $1026 and $4035), upper middle-income countries (GNI per capita between $4036 and $12,475), and high-income countries (GNI per capita of $12,476 or more) (Barbados and Singapore) [27]. Confounding factors, selected based on literature review [6,7,8,9,10], included sociodemographic and lifestyle factors, including psychiatric morbidity, i.e. depression. Study countries were grouped into four regions according to epidemiological similarly and geographic proximity. The degree of missing values across the variables was generally less than 2.5% (2.0% for sleep duration, 1.8% for sleep quality, 0 for BMI, and 4.4% for WC). Missing values were excluded from the analysis. Potential multi-collinearity between variables was assessed with variance inflation factors, none of which exceeded critical value. *p* < 0.05 was considered significant. The country was entered as the primary sampling unit for survey analysis in STATA in order to achieve accurate Confidence Intervals (CIs), given the clustered nature of the data.

## 3. Results

### 3.1. Sample Characeristics

The sample included 18,211 (42.1% male and 57.9% female, mean age 21.0 years in male and 20.7 years in female students, range 16–30 years) undergraduate university students; 2742 from Central Latin America and the Caribbean, 4866 from sub-Saharan Africa, 7293 from South and Southeast Asia, and 3310 from Central Asia, North Africa, the Middle East and Eastern Europe (=Central Asia and other regions). Of the study sample, 9725 participants were from low- or lower middle-income and 8486 were from upper middle- or high-income countries. The overall BMI was a mean of 22.5 kg/m^2^ (SD = 4.5) for men and 22.0 kg/m^2^ (SD = 4.7) for women, and the mean WC was 78.4 cm (SD = 11.5) for men and 73.8 cm (SD = 11.4) for women. Among men and women, the BMI was the highest in the Central Latin American and Caribbean region (24.0, SD = 4.6 and 23.4, SD = 5.3, respectively), and among men the WC was the highest in the Central Asia, North Africa, Middle East and Eastern European region (83.1, SD = 11.5), while among women it was the highest in the Central Latin American and Caribbean region (77.1, SD = 11.9). Almost two in five of the students (39.4%) reported short sleep duration and 32.7% reported moderate to extreme sleeping problems.

Table 1 describes sleep duration and sleep quality by sociodemographic and health risk behaviour variables. The prevalence of having a sleep problem was higher in older and wealthier students, and students from Central Latin America and the Caribbean had the highest prevalence of short sleep compared to the other regions. The prevalence of short sleep was lower among current tobacco users, while the prevalence of poor sleep was higher among current tobacco users. Students with depressive symptoms had a higher prevalence of short sleep and poor sleep quality.

### 3.2. Association between Sleep Duration, Sleep Quality, and BMI by Gender

In a linear multivariable regression, adjusted for sociodemographic and lifestyle factors, short sleep duration was positively associated with BMI in both men (Regression Coefficient (B) = 0.36, 95% CI = 0.07, 0.65) and women (B = 0.40, 95% CI = 0.13, 0.68). Among men, sleep quality or problems were not associated with BMI, while among women mild sleep problems were inversely associated with BMI. Further, coming from the Central Latin America and the Caribbean was positively associated with BMI (see Table 2).

### 3.3. Association between Sleep Duration, Sleep Quality, and WC by Gender

In a linear multivariable regression, adjusted for sociodemographic and lifestyle factors, short sleep duration was positively associated with WC among women (B = 1.16, 95% CI = 0.45, 1.86), but not among men. Among both men and women, poor sleep quality or problems were positively associated with WC (B = 1.46, 95% CI = 0.45, 2.47, and B = 1.34, 95% CI = 0.11, 2.57, respectively). Further, coming from Central Latin America and the Caribbean was positively associated with WC (see Table 3).

### 3.4. Association between Sleep Duration, Sleep Quality, BMI, and WC by Country Income

In a linear multivariable regression, adjusted for sociodemographic and lifestyle factors, short sleep duration was positively associated with BMI in both students from low- or lower middle-income countries (B = 0.32, 95% CI = 0.13, 0.51) and in students from upper middle- or high-income countries (B = 0.25, 95% CI = 0.03, 0.46). Among both students from low- or lower middle-income countries and in students from upper middle- or high-income countries, severe or extreme sleep quality or problems were not associated with BMI.

In a linear multivariable regression, adjusted for sociodemographic and lifestyle factors, short sleep duration was positively associated with WC in students from low- or lower middle-income countries (B = 1.03, 95% CI = 0.31, 1.75), but not in students from upper middle- or high-income countries. Among students from upper middle- or high-income countries, severe or extreme sleep quality or problems were positively associated with WC (B = 2.66, 95% CI = 1.27, 4.04), but not in students from low- or lower middle-income countries (see Table 4).

### 3.5. Association between Sleep Duration, Sleep Quality, BMI, and WC by Study Region

In a linear multivariable regression, adjusted for sociodemographic and lifestyle factors, short sleep duration was positively associated with BMI (B = 0.43, 95% CI = 0.003, 0.86, and B = 0.41, 95% CI = 0.19, 0.64) and WC (B = 1.83, 95% CI = 0.78, 2.89, and B = 1.22, 95% CI = 0.61, 1.82) among students from Central Latin America and Caribbean, and South or Southeast Asia, respectively. Among students from sub-Saharan Africa, mild and severe or extreme sleep problems were negatively associated with BMI. Further, among students from Central Asia and neighbouring regions, short sleep duration was negatively and having sleep problems was positively associated with WC (see Table 5).

## 4. Discussion

This study examined the relationship between sleep duration, quality, BMI, and WC in young adults in low- and middle-income countries. Previous research focused on investigating these variables predominantly in high-income countries, and found evidence that short sleep duration and poor sleep quality was associated with increased BMI and WC [8,9,10,11,28]. This study confirmed an association between short sleep duration and increased BMI and, among women only, increased WC. This gender difference is not consistent with previous research [29]. So, our findings may add to some of the other research studies proposing that sleep duration might have a greater impact on increased WC in women than in men [30]. Further research is needed to explore the potential gender differences, as several studies found similar or opposite gender differences in the relationship between sleep duration and increased BMI [30,31].

An association between poor sleep quality and increased WC but not increased BMI was found, which is confirmed in a systematic review [9]. This difference between increased BMI and WC may be related to the different mechanisms at play with each type of obesity [32]. A pattern of poor sleep quality may lead to changes in appetite regulation and “thus influence food choice and calorie intake” [33] Long sleep duration was not associated with increased BMI or WC in this study, as has been found in several previous studies, including a large Thai open-university sample [29] and a sample of black and white US adults [34]. Thus, this study does not support a U-shaped association between sleep duration and BMI and WC [31].

Further, students from Central Latin America and the Caribbean in this study were more likely to be short sleepers and had an increased BMI and WC, relative to their counterparts from the other three study regions (sub-Saharan Africa, South/Southeast Asia, and Central Asia and neighbouring regions). The mean BMI (23.7) and the proportion of short sleepers (42.3%) were the highest in the Central Latin American and Caribbean region, compared to the other three regions. Considering these regional differences, the regions were analysed separately, resulting in a positive association between short sleep and BMI and WC in the Central Latin American and Caribbean and the South/Southeast Asia regions, but not in the sub-Saharan Africa and the Central Asia and other regions. This regional difference could mean that a geographic region should be considered in the analysis of data linking short sleep and BMI and WC [34]. An analysis conducted separately for low- or lower middle-income countries and upper middle- or high-income countries found an association between short sleep and WC in low- or lower-middle income countries and between poor sleep quality and WC in upper middle- or high-income countries. This finding shows differences between sleep characteristics and WC by country income, which calls for further explorative studies to identify such differences.

The study showed a high rate of abnormal sleep, in particular short sleep, and poor sleep quality, which significantly linked with obesity, suggesting the need to expand health promotion programmes to encourage healthy sleeping, eating, and physical activity in this population [35].

### Study Limitations

This study had several limitations. The investigation was carried out with students from one university in each country, mostly in urban centres, and the inclusion of other centres could have resulted in different results. University students are not representative of young adults in general, and the BMI and WC and its associated factors may be different in other sectors of the population. The study was a cross-sectional study and the temporal relationships between sleep practices and obesity cannot be established in such studies; further longitudinal studies are needed. Apart from anthropometric measurements, a limitation of the study was that all the other information, including sleep characteristics, was collected based on self-reporting. Compared with objective methods, self-reported sleep duration tends to overreport sleep, which is at a higher rate among short sleepers than long sleepers [36]. This could mean that in this study a larger proportion of short sleepers would have been found with the use of objective sleep measures. Future studies may consider more reliable methods such as the use of sleep diaries [10]. Wener et al. [37] found that differences between actigraphy and diary had a satisfactory agreement between methods (+/− 32 min for assumed sleep), while the agreement rates between actigraphy and questionnaire as well as between diary and questionnaire were insufficient. Further, the grouping of our study country has its limitations, in particular because three regions, Central Asia, North Africa and the Middle East, and Eastern Europe, were grouped together.

## 5. Conclusions

This study found a significant association between short sleep duration and increased BMI and, among women, increased WC, as well as an association between poor sleep quality and increased WC but not increased BMI among university students across 26 low- and middle-income countries. Further, differences in the association between sleep characteristics and BMI and WC were found by region and country income. This information may help in obesity prevention programmes.

## Figures and Tables

**Table 1:** Sleep duration and quality by independent variables (*n =* 18,211).

Variable	*n*	Sleep Duration (*n =* 17845) ^1^		Sleep Quality-Problem (*n =* 17875) ^2^		
		6 or Less	9 or More	Mild	Moderate	Severe/Extreme
		M (SD)	M (SD)	M (SD)	M (SD)	M (SD)
**Sociodemographics**						
Age (in years)	18,189	21.0 (3.0)	21.0 (2.8)	20.7 (2.7)	21.0 (2.8)	21.1 (3.0)
**Socioeconomic Status**						
Poor	9201	39.4	13.1	34.9	25.4	8.8
Wealthy	8909	40.0	13.8	30.6	24.7	9.1
**Region**						
Central Latin America/Caribbean	2742	42.3	16.1	32.1	26.7	10.2
Sub-Saharan Africa	4866	39.3	16.2	26.1	28.9	11.0
South/Southeast Asia	9725	39.1	11.9	36.3	23.6	6.8
Central Asia and other regions	3310	36.6	14.6	31.4	20.3	10.8
**Country Income**						
Low- or lower middle-income ^3^	9725	37.7	13.5	31.0	24.8	9.3
Upper middle- or high-income ^4^	8486	41.5	13.4	34.3	25.3	8.7
**Health Risk Behaviours**						
Tobacco use						
No	15,848	39.7	12.8	33.9	24.7	8.1
Yes	2140	36.5	17.5	27.9	25.1	14.6
Binge alcohol use						
No	14,140	40.1	12.6	32.5	24.5	8.6
Yes	3758	38.4	16.2	33.5	26.9	10.2
Physical inactivity						
No	10,574	41.0	12.0	33.1	25.4	8.8
Yes	7348	40.0	13.0	35.7	23.5	8.3
Sedentary behaviour						
No	12,378	40.7	13.2	32.9	26.8	8.6
Yes	5509	43.1	12.2	33.9	25.2	9.0
Depressive symptoms						
No	15,834	39.0	13.2	33.7	24.1	7.1
Yes	2366	44.4	15.0	26.6	31.3	21.6

^1^ Reference category for sleep duration is normal sleep (7–8 h); ^2^ Reference category for sleep quality-problem is “None”; ^3^ Low- or lower middle-income country: Bangladesh, India, Ivory Coast, Kenya, Kyrgyzstan, Laos, Madagascar, Myanmar, Nigeria, Pakistan, Philippines, Tunisia and Vietnam; ^4^ Upper middle- or high-income country: Barbados, Colombia, Grenada, Jamaica, Malaysia, Mauritius, Namibia, Russia, Singapore, South Africa, Thailand, Turkey, and Venezuela [27]; M: Mean, SD: Deviation

**Table 2:** Multivariable linear regression models for predictors of Body Mass Index (BMI) by gender.

Variable	Men		Women	
	Unadjusted	Adjusted ^1^	Unadjusted	Adjusted ^1^
	Regression Coefficient (95% CI)	Regression Coefficient (95% CI)	Regression Coefficient (95% CI)	Regression Coefficient (95% CI)
**Sleep Duration in Hours**				
7–8	Reference	Reference	Reference	Reference
6 or less	0.24 (0.03, 0.45) *	0.36 (0.07, 0.65) *	0.28 (0.07, 0.48) **	0.40 (0.13, 0.68) **
9 or more	0.25 (−0.05, 0.56)	0.18 −0.25, 0.63)	0.25 (−0.03, 0.54)	0.02 (−0.38, 0.41)
**Region**				
Central Latin				
America/Caribbean		Reference		Reference
Sub-Saharan Africa		−2.31 (−2.74, −1.87) ***		−0.70 (−1.11, −0.28) ***
South/Southeast Asia		−1.46 (−1.84, −1.08) ***		−2.03 (−2.35, −1.71) ***
Central Asia and other regions		−0.32 (−0.89, 0.25)		−1.76 (−2.32, −1.21) ***
**Sleep Quality-Problem**				
None	Reference	Reference	Reference	Reference
Mild	−0.11 (−0.35, 0.14)	−0.40 (−0.73, 0.07)	−0.37 (−0.61, −0.14) **	−0.37 (−0.69, −0.07) *
Moderate	−0.10 (−0.36, 0.16)	−0.18 (−0.54, 0.19)	−0.05 (−0.30, 0.20)	−0.11 (−0.45, 0.19)
Severe or Extreme	0.29 (−0.09, 0.66)	−0.13 (−0.66, 0.40)	0.12 (−0.23, 0.47)	0.004 (−0.49, 0.40)
**Region**				
Central Latin America/Caribbean		Reference		Reference
Sub-Saharan Africa		−2.30 (−2.74, −1.87) ***		−0.72 (−1.13, −0.30) ***
South/Southeast Asia		−1.48 (−1.86, −1.10) ***		−2.04 (−2.36, −1.72) ***
Central Asia and other regions		−0.38 (−0.95, −1.87) ***		−1.82 (−2.37, −1.26) ***

^1^ Adjusted for sociodemographic factors (age socioeconomic status) and lifestyle factors (tobacco use, binge alcohol use, physical inactivity, sedentary behaviour, and depressive symptoms); *** *p* < 0.001; ** *p* < 0.01; * *p* < 0.05.

**Table 3:** Multivariable linear regression models for predictors of waist circumference (WC) by gender.

Variable	Men		Women	
	Unadjusted	Adjusted ^1^	Unadjusted	Adjusted ^1^
	Regression Coefficient (95% CI)	Regression Coefficient (95% CI)	Regression Coefficient (95% CI)	Regression Coefficient (95% CI)
**Sleep Duration**				
7–8 hrs	Reference	Reference	Reference	Reference
6 or less	0.35 (−0.21, 0.92)	0.60 (−0.19, 1.39)	0.96 (0.45, 1.46) ***	1.16 (0.45, 1.86) ***
9 or more	−0.08 (−0.89, 0.73)	−0.04 (−1.21, 1.13)	0.85 (0.15, 1.55) *	−0.02 (−1.05, 1.01)
**Region**				
Central Latin America/Caribbean		Reference		Reference
Sub-Saharan Africa		−6.54 (−7.68, −5.39) ***		5.54 (−4.42, −2.36) ***
South/Southeast Asia		−3.51 (−5.54, −2.48) ***		−6.02 (−6.85, −5.19) ***
Central Asia and other regions		7.55 (6.06, 9.03) ***		−1.47 (−2.29, −0.65) ***
**Sleep Quality-Problem**				
None	Reference	Reference	Reference	Reference
Mild	−0.02 (−0.66, 0.63)	−0.56 (−1.46, 0.33)	−0.26 (−0.84, 0.33)	−0.34 (−1.18, 0.49)
Moderate	−0.26 (−0.94, 0.42)	−0.30 (−1.27, 0.67)	0.66 (0.05, 1.27) *	0.71 (−0.15, 1.57)
Severe or Extreme	1.60 (0.63, 2.58) ***	1.17 (0.10, 2.25) *	1.38 (0.53, 2.24) ***	1.34 (0.11, 2.57) *
**Region**				
Central Latin America/Caribbean		Reference		Reference
Sub−Saharan Africa		−6.41 (−7.56, −5.27) ***		−3.38 (−4.41, −2.35) ***
South/Southeast Asia		−3.49 (−4.51, −2.36) ***		−6.10 (−6.92, −5.27) ***
Central Asia and other regions		3.49 (2.21, 4.77) ***		−1.51 (−2.33, −0.70) ***

^1^ Adjusted for sociodemographic factors (age socioeconomic status) and lifestyle factors (tobacco use, binge alcohol use, physical inactivity, sedentary behaviour, and depressive symptoms); *** *p* < 0.001; ** *p* < 0.01; * *p* < 0.05.

**Table 4:** Multivariable linear regression models for predictors of BMI and WC by country income.

Variable	Low- or Lower Middle-Income Country		Upper Middle- or High-Income Country	
	Unadjusted	Adjusted ^1^	Unadjusted	Adjusted ^1^
	Regression Coefficient (95% CI)	Regression Coefficient (95% CI)	Regression Coefficient (95% CI)	Regression Coefficient (95% CI)
**BMI**				
**Sleep Duration**				
7–8 h	Reference	Reference	Reference	Reference
6 or less	0.32 (0.13, 0.51) ***	0.30 (0.11, 0.50) **	0.31 (0.13, 0.51) **	0.25 (0.03, 0.46) *
9 or more	0.13 (−0.14, 0.40)	0.11 (−0.16, 0.39)	0.21 (−0.10, 0.52)	0.24 (−0.07, 0.54)
**Sleep Quality-Problem**				
None	Reference	Reference	Reference	Reference
Mild	−0.17 (−0.40, 0.07)	−0.48 (−0.82, −0.15) **	−0.53 (−0.77, −0.28) ***	−0.28 (−0.61, 0.04)
Moderate	−0.25 (−0.50, −0.002) *	−0.53 (−0.88, −0.18) **	−0.05 (−0.31, 0.21)	0.27 (−0.09, 0.62)
Severe or Extreme	0.26 (−0.10, 0.62)	−0.42 (−0.94, 0.10)	−0.03 (−0.39, 0.34)	0.41 (−0.10, 0.91)
**WC**				
**Sleep Duration**				
7−8 hrs	Reference	Reference	Reference	Reference
6 or less	1.01 (0.52, 1.50) ***	1.03 (0.31, 1.75) **	−0.08 (−0.67, 0.51)	−0.29 (−1.08, 0.51)
9 or more	0.56 (−0.12, 1.24)	0.16 (−0.97, 1.28)	0.13 (−0.69, 0.95)	0.30 (−0.80, 1.40)
**Sleep Quality-Problem**				
None	Reference	Reference	Reference	Reference
Mild	−0.76 (−1.32, −0.21) **	−1.19 (−2.03, −0.35) **	−0.46 (−1.14, 0.22)	0.27 (−0.64, 1.18)
Moderate	−0.53 (−1.12, 0.06)	−0.75 (−1.64, 0.14)	0.09 (−0.62, 0.80)	0.80 (−0.18, 1.77)
Severe or Extreme	0.58 (−0.26, 1.42)	−0.43 (−1.75, 0.89)	1.54 (0.54, 2.53) **	2.66 (1.27, 4.04) ***

^1^ Adjusted for sociodemographic (age, gender, socioeconomic status) and lifestyle factors (tobacco use, binge alcohol use, physical inactivity, sedentary behaviour, and depressive symptoms); *** *p* < 0.001; ** *p* < 0.01; * *p* < 0.05.

**Table 5:** Multivariable linear regression models for predictors of BMI and WC by study region.

Variable	Central Latin America/Caribbean	Sub-Saharan Africa	South/Southeast Asia	Central Asia and Others
	Adjusted ^1^	Adjusted ^1^	Adjusted ^1^	Adjusted ^1^
	Regression Coefficient (95% CI)	Regression Coefficient (95% CI)	Regression Coefficient (95% CI)	Regression Coefficient (95% CI)
**BMI**				
**Sleep Duration**				
7–8 h	Reference	Reference	Reference	Reference
6 or less	0.43 (0.003, 0.86) *	0.16 (−0.26, 0.57)	0.41 (0.19, 0.64) ***	−0.29 (−0.60, 0.02)
9 or more	−0.04 (−0.59, 0.51)	0.31 (−0.28, 0.90)	−0.02 (−0.38, 0.35)	0.06 (−0.40, 0.51)
**Sleep Quality-Problem**				
None	Reference	Reference	Reference	Reference
Mild	0.08 (−0.40, 0.56)	−0.66 (−1.16, −0.16) **	−0.40 (−0.65, −0.14) **	0.14 (−0.21, 0.49)
Moderate	0.53 (0.01, 1.04) *	−0.49 (−0.99, 0.02)	−0.08 (−0.36, 0.20)	0.07 (−0.33, 0.48)
Severe or Extreme	0.56 (−0.17, 1.28)	−0.80 (−1.50, −0.10) *	0.12 (−0.33, 0.56)	0.38 (−0.11, 0.87)
**WC**				
**Sleep Duration**				
7–8 h	Reference	Reference	Reference	Reference
6 or less	1.83 (0.78, 2.89) ***	−0.40 (−1.36, 0.55)	1.22 (0.61, 1.82) ***	−2.30 (−3.40, −1.21) ***
9 or more	−0.26 (−1.61, 1.09)	0.82 (−0.53, 2.17)	−0.04 (−1.03, 0.96)	0.32 (−1.28, 1.93)
**Sleep Quality-Problem**				
None	Reference	Reference	Reference	Reference
Mild	−0.13 (−1.32, 1.07)	−0.03 (−1.19, −1.13)	−1.01 (−1.70, −0.31) **	2.64 (1.41, 3.86) ***
Moderate	1.16 (−1.16, 2.44)	0.12 (−1.04, 1.27)	−0.28 (−1.03, 0.47)	2.39 (0.96, 3.82) ***
Severe or Extreme	0.11 (−0.35, 3.25)	0.07 (−1.54, 1.68)	0.47 (−0.72, 1.65)	3.36 (1.63, 5.10) ***

^1^ Adjusted for sociodemographic (age, gender, socioeconomic status) and lifestyle factors (tobacco use, binge alcohol use, physical inactivity, sedentary behaviour, and depressive symptoms); *** *p* < 0.001; ** *p* < 0.01; * *p* < 0.05.
